# Wound Morphology and Topography in the Diabetic Foot: Hurdles in Implementing Angiosome-Guided Revascularization

**DOI:** 10.1155/2014/672897

**Published:** 2014-02-02

**Authors:** Dimitri Aerden, Nathalie Denecker, Sarah Gallala, Erik Debing, Pierre Van den Brande

**Affiliations:** ^1^Diabetic Foot Clinic, Universitair Ziekenhuis Brussel, Laarbeeklaan 101, 1090 Jette, Belgium; ^2^Department of Vascular Surgery, Universitair Ziekenhuis Brussel, Laarbeeklaan 101, 1090 Jette, Belgium

## Abstract

*Purpose*. Angiosome-guided revascularization is an approach that improves wound healing but requires a surgeon to determine which angiosomes are ischemic. This process can be more difficult than anticipated because diabetic foot (DF) wounds vary greatly in quantity, morphology, and topography. This paper explores to what extent the heterogeneous presentation of DF wounds impedes development of a proper revascularization strategy. *Methods*. Data was retrieved from a registry of patients scheduled for below-the-knee (BTK) revascularization. Photographs of the foot and historic benchmark diagrams were used to assign wounds to their respective angiosomes. *Results*. In 185 limbs we detected 345 wounds. Toe wounds (53.9%) could not be designated to a specific angiosome due to dual blood supply. Ambiguity in wound stratification into angiosomes was highest at the heel, achilles tendon, and lateral/medial side of the foot and lowest for malleolar wounds. In 18.4% of the DF, at least some wounds could not confidently be categorized. Proximal wounds (coinciding with toe wounds) further steered revascularization strategy in 63.6%. Multiple wounds required multiple BTK revascularization in 8.6%. *Conclusion*. The heterogeneous presentation in diabetic foot wounds hampers unambiguous identification of ischemic angiosomes, and as such diminishes the capacity of the angiosome model to optimize revascularization strategy.

## 1. Introduction

Below-the-knee (BTK) revascularization encompassing endovascular angioplasty and distal bypass surgery is essential for successful treatment of ischemic diabetic foot ulcers [1]. Angiosome-guided revascularization is a paradigm that has generated considerable interest since studies have suggested that direct revascularization of the appropriate angiosome (where antegrade pulsatile flow is reinstated to the angiosome that harbors the ulcer) yields superior results compared to indirect revascularization [2, 3]. On the contrary, some authors have disputed that angiosome-guided revascularization considerably improves clinical outcome [4–7].

Angiosome-guided revascularization implies that the decision of which artery to target for revascularization is based on where the ulcer is located. Ideally, there should be no doubt as to which angiosome requires reperfusion so that the surgeon can focus exclusively on opening those arteries that are indispensable for wound healing. In practice, however, diabetic patients present with a multitude of wounds that are heterogeneous in morphology and topography. For example, a patient may present with several wounds dispersed over more than one angiosome or manifest a large ulcer that lies on the verge of two angiosomes. Under these circumstances, determining which below-the-knee artery to target for revascularization may be less straightforward than anticipated.

In this study, we assessed the localization and morphology of ischemic diabetic foot wounds. Based on the presentation of these wounds, we set out to investigate the level of difficulty to identify which angiosomes require revascularization.

## 2. Materials and Methods

A prospective registry of diabetic foot patients has been established in 2004 at our diabetic foot clinic, in a university hospital. This registry contains general demographic data as well as digital photographs of the wounds that patients initially present with. These high-resolution pictures are taken from multiple angles so that all sides of the foot are recorded. From this database, we retrieved all patients that had been scheduled for BTK revascularization. The indication for revascularization was established when the angiography showed at least one high-grade stenosis (>50% lumen diameter reduction) or occlusion distal from the popliteal artery. The localisation and shape of each wound was discerned from photographs, and we tried to determine to which angiosome a wound belonged. The anatomical demarcation of foot angiosomes was obtained from benchmark diagrams previously sketched up by Taylor et al. [8] and Attinger et al. [9]. If we were unable to unambiguously assign a wound to its angiosome, we recorded the reason why. Two investigators independently scrutinized the pictures and if discrepancies were found, the mismatched cases were reassessed and resolved by consensus.

Vascular specialists critical of the angiosome model do not take into account wound localization when selecting an artery for treatment, but instead prefer to be guided by technical revascularization feasibility or distal outflow. For this reason, we also assessed atherosclerotic disease by studying the intra-arterial angiographies: each below-the-knee artery as well as the tibioperoneal trunk was scored for its hemodynamically most significant lesion (according to the Joint Vascular Societies reporting standard [10]).

## 3. Results

**Figure 1:**
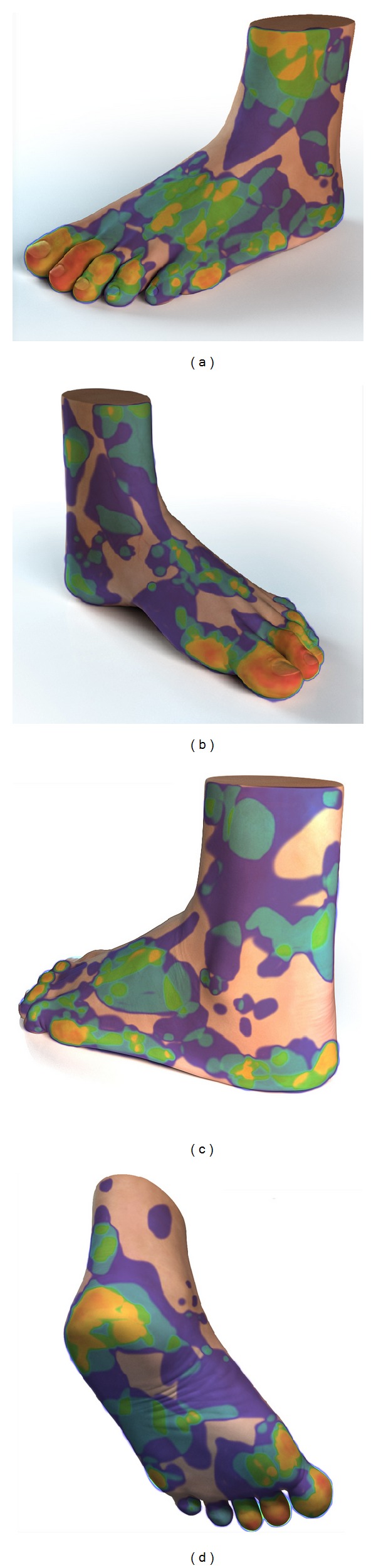
Composite image of all wounds showing predisposing areas. Likelihood to contain wounds varies from red (most likely) to blue (least likely).

Hundred and eighty-five (*n* = 185) diabetic feet harboring 345 distinctive wounds were included in the study (see demographic data in Table 1). Almost half of the patients presented with a single wound defect, whereas the remainder showed multiple wounds in different locations. To identify locations that predisposed to the development of wounds, we traced the outline of each wound onto a standard foot diagram. All individual wound shapes were then superimposed to create a single composite image (Figure 1). This image showed a high predilection for the toes and metatarsal heads (predominantly of the first and second ray), as well as pressure points such as the medial side of the first metatarsal head and the lateral side of the fifth metatarsal head. The heel and base of the fifth metatarsal head were frequently involved as well.

**Figure 2:**
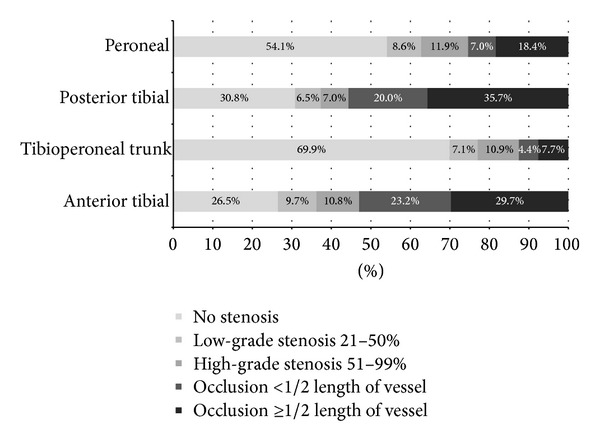
Distribution of atherosclerotic lesions on angiography (*n* = 185).

Figure 2 shows how atherosclerotic disease was distributed. The tibial arteries were heavily affected by atherosclerosis, showing occlusions in half of the cases. The three below-the-knee arteries were equally diseased (had the same score) in 7.6% of the cases, which means that technically all three arteries would be equally suitable for revascularization. We determined if lower extremities had one (*n* = 73 or 39.5%), two (*n* = 65 or 35.1%), or all three (*n* = 15 or 8.1%) BTK arteries occluded, while an absence of any occlusions implicated that arteries were affected by high-grade stenosis exclusively (*n* = 32 or 17.3%). Finally, we compared the degree of atherosclerosis in both tibial arteries because these arteries become the dorsal and plantar arc of the foot: only 23.8% of all angiographies showed comparable occlusive disease in these arteries. Assessment of these angiographies suggests that revascularization feasibility differs considerably between BTK arteries in the majority of the cases.

In the diabetic foot, a comprehensive assessment of all residing ulcers is required to devise a proper revascularization strategy. The diagrams that depict anatomically demarcated angiosomes clearly show which areas are supplied by each of the three BTK arteries. Connecting a wound to its feeding artery is straightforward for proximal wounds but proves problematic in toe wounds that receive dual blood supply from both the dorsal and plantar arterial arc. This anatomical setup makes discrimination between the anterior or posterior tibial artery as the feeding artery of toe wounds impossible, a notion that is highly relevant since toe wounds were so predominant (49.0%) in our population. Consequently, wounds distal to the metatarsal arc (toe wounds, nonhealing amputation sites, and wounds at the interdigital web space) were grouped together as these would imply opening up either one of the tibial arteries.

Classification of proximal wounds proved ambiguous in 23.3%, mainly because of wounds located at the heel or the lateral side of the foot. In these regions, wounds often extended into adjacent angiosomes (decubitus wounds) or were located on the verge of two bordering angiosomes (where the plantar and dorsal part of the foot collides). In addition, we could not derive from the artist's rendition of angiosomes in several charts whether the achilles tendon was supplied by the peroneal or tibial posterior artery. Wounds at the ankle could be classified most confidently, as these wounds were small and predominantly confined to the bony prominence of the malleoli, a territory that is undisputedly linked to the peroneal or tibial anterior artery.  Table 2 shows how individual wounds were categorized.

**Figure 3:**
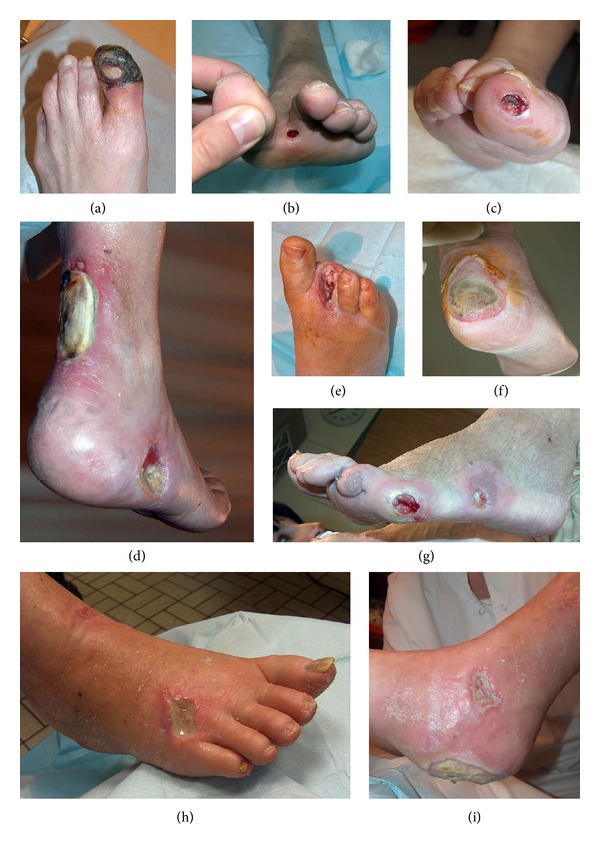
Examples of wounds with ambiguous angiosome categorization. (a), (b), (c), (e) Circumferential necrosis, wounds located at the tip or interdigital webspace, and nonhealing amputation sites do not allow differentiation between angiosomes derived from the anterior and posterior tibial artery. (d) Territories such as the achilles tendon are not equivocally associated with a particular angiosome. (f), (g) Wounds that extend into an adjacent angiosome, or lie on the verge of two angiosomes. (h), (i) Two pictures of the same foot showing multiple wounds (dorsal, medial malleolar, and heel) residing in different angiosomes.

The assessment of individual wounds is less relevant than the appraisal of the diabetic foot as a whole.  Table 3 shows how wound composition differed between diabetic feet and how this reflected on revascularization planning. Eighty-five limbs (*n* = 85 or 45.7%) contained only toe wounds, which implies revascularization of either the anterior or posterior tibial artery. The remainder harbored additional proximal wounds (*n* = 33 or 17.7%) or manifested proximal wounds exclusively (*n* = 67 or 36.0%). The presence of proximal wounds elicited additional guidance towards revascularization provided that they could be categorized unambiguously into specific angiosomes. When proximal wounds coincided with toe wounds, we found further arguments to discriminate between tibial arteries or additional targeting of the peroneal artery in 63.6% of the cases. Likewise, analysis of cases with only proximal wounds suggested targeting of a single BTK artery in 73.1% and two or more BTK arteries in 17.9%. The level of uncertainty in both groups was manifested by the fact that in 34 cases (18.4%) at least some of the wounds could not confidently be categorized. In 8.6% of all limbs, the presence of multiple wounds dispersed over several angiosomes suggested revascularization of more than one BTK artery.  Figure 3 shows pictures of wounds that for a variety of reasons were difficult to assign to a specific angiosome.

## 4. Discussion

The angiosome model is a very intuitive concept that is easy to endorse: no vascular specialist will dispute that restoring pulsatile arterial flow to an ischemic block of tissue promotes healing. However, it remains unclear to what extent the angiosome model influences revascularization strategy and decision-making that significantly improves clinical outcome. Indeed, critics of the angiosome model claim that indiscriminate recanalization of any tibial artery that restores perfusion to the plantar or dorsal arc of the foot will likewise accomplish wound healing, regardless of where the wound is located [5].

The ability of the angiosome model to steer decision-making is particularly relevant in conditions where an interventionist is forced to target one single artery, for example, in bypass surgery or in an attempt to limit contrast-load in patients with end-stage kidney failure. However, the endovascular-first approach advocated for diabetic foot allows sequential revascularization of all arteries, which means that the choice of which artery to target first becomes less stringent and renders the angiosome model less determinant.

Our study was not designed to question or validate the angiosome model per se. Regardless, it may be important to note that Taylor and Attinger delineated angiosomes in cadaver limbs devoid of arterial occlusive disease. In such limbs unaffected by atherosclerosis, a complex redundant network of collaterals can be recruited to protect tissue viability when one particular BTK artery occludes. Conversely, this compensatory system is compromised in diabetic patients, which renders distal feeding arteries into functional end arteries [11, 12]. As a result, ischemic tissue blocks in the diabetic foot may not overlap completely with the anatomical angiosomes mapped out by Taylor and Attinger. It remains unclear to what extent a compensatory system (or lack thereof) should be regarded as a confounding factor in angiosome-guided revascularization. Finally, the landmark studies of the angiosome model did not take into account the many anatomical variances of BTK and foot arteries [13]: in some instances, the feeding artery of an angiosome may indeed be derived from a different BTK artery than standard angiosome-sketches suggest. Only a peroperative angiographic assessment with attention to anatomical variants and collateral flow (as opposed to a pure anatomical topographic classification) reliably confirms which artery should be considered as the feeding artery of a wound bed.

Our study shows that diabetic foot patients present with a variety of wounds of very diverse morphology and localization. Based on the presentation of these wounds, we found that in many cases it was anything but straightforward to decide which BTK artery to target first for revascularization. In addition to the aforementioned reservations, this may be an important hurdle in the implementation of the angiosome model.

However, our study certainly has a number of limitations. One obvious shortcoming is that no outcome data was analyzed: it would have been interesting to see if unambiguously classified wounds fared better after direct revascularization than hard-to-classify wounds. Unfortunately, no reliable outcome data was at our disposal. Wound etiology (infection, bone involvement, neuropathy) and followup (wound healing, minor amputation) were insufficiently documented, while revascularization strategy lacked standardization (open and endovascular procedures as well as simultaneous treatment of above-the-knee or other BTK-arteries).

Second, we have suggested that wound topography does not always allow for unequivocal identification of the BTK artery that should receive priority for revascularization. However, one could argue that the potential to exclude an artery from treatment (narrowing down options to two arteries) is also beneficial.

Third, the mere presence of a wound is only indicative of ischemia in an angiosome. In practice, additional tests like TCpO_2_ measurements, Doppler analysis, and peroperative selective angiography all aid to determine if an angiosome is truly ischemic; no such tests were evaluated in our study.

## 5. Conclusion

We believe that wound localization and morphology in the diabetic foot do not invariably allow unambiguous identification of angiosomes that are ischemic and require revascularization. This finding may limit the capacity of the angiosome model to optimize revascularization strategy; additional studies are required to ascertain if other factors, such as technical feasibility, inherent to the type, and distribution of atherosclerotic disease, are more determinant.

## Figures and Tables

**Table 1:** Diabetic feet with peripheral arterial disease (*n* = 185).

Age (years)	71.8 ± 10.3	
Gender (male/female)	123 (66.5%)	62 (33.5%)
Side (right/left)	100 (54.1%)	85 (45.9%)
One single foot wound	92 (49.7%)	
Two wounds	44 (23.8%)	
Three or more	49 (26.5%)	

	185 (100.0%)	

**Table 2:** Categorization of individual wounds into angiosomes (*n* = 345).

Toe wounds (grouped)			
Toe wounds (including webspace)	169 (49.0%)		
Toe amputation sites	16 (4.6%)	No classification into angiosome possible (either tibial artery is elible for revascularization)	
Forefoot amputation site	1 (0.3%)		
	**186 (53.9%)**		

Proximal wounds		Classification into angiosome	
		Unambiguous	Ambiguous

Plantar foot (excluding the heel)	25 (7.2%)	19 (76.0%)	6 (24.0%)
Dorsal foot	23 (6.7%)	21 (91.3%)	2 (8.7%)
Lateral or medial side of the foot	43 (12.5%)	25 (58.1%)	18 (41.9%)
Heel (plantar, lateral, and medial)	23 (6.7%)	17 (73.9%)	6 (26.1%)
Ankle (malleolar)	23 (6.7%)	23 (100.0%)	0 (0.0%)
Above the ankle	22 (6.4%)	17 (77.3%)	5 (22.7%)
	**159 (46.1%)**	**122 (76.7%)**	**37 (23.3%)**
Total	**345 (100.0%)**		

**Table 3:** Wound composition in diabetic feet (*n* = 185).

Wound composition			Revascularization strategy
Feet with toe wounds exclusively	85 (45.9%)		85 anterior or posterior tibial artery revascularization
Feet with toe wounds and proximal wounds	33 (17.8%)		
Wounds that could be unambiguously classified			
All Some	16 (8.6%) 5 (2.7%)	=21	2 additional peroneal artery revascularisation 14 additional argument for anterior tibial revascularisation 3 additional argument for posterior tibial revascularisation 2 proximal wounds suggest treatment of both tibial arteries
None	12 (6.5%)		?
Feet with proximal wounds exclusively	67 (36.2%)		
Wounds that could be unambiguously classified			
All	50 (27.0%)	=61	49 revascularization of a single BTK artery
Some	11 (5.9%)		12 revascularization of two BTK arteries
None	6 (3.2%)		?
